# Endodontic Management of Maxillary Second Molar with Two Palatal Roots: A Report of Two Cases

**DOI:** 10.1155/2012/590406

**Published:** 2012-12-04

**Authors:** Surbhi Patel, Pawan Patel

**Affiliations:** ^1^Department of Conservative Dentistry & Endodontics, MGV's Karmaveer Bhausaheb Hire Dental College, Nashik 422003, India; ^2^Department of Conservative Dentistry & Endodontics, Yogita Dental College, Khed, Ratnagiri 415709, India

## Abstract

Endodontic treatment may sometimes fail because morphological features of the tooth adversely affect the treatment protocol. Maxillary second molars are recognized as usually having a single palatal root with a single palatal canal. The incidence of second palatal root in the maxillary second molar is very rare. Two cases are presented in this paper describing the endodontic management of a four-rooted maxillary second molar with two distinct palatal roots and canals and two distinct buccal roots and canals. Clinical examination and radiographs showed the presence of two palatal roots during the root canal procedure. The canals were biomechanically prepared with crown-down technique and obturated using lateral condensation technique with AH-Plus sealer.

## 1. Introduction

The main objective of root canal treatment is the thorough mechanical and chemical debridement of the entire pulp space followed by complete obturation with an inert filling material. Therefore, it is imperative that aberrant anatomy is identified prior to and during root canal treatment. Careful evaluation of research material has, however, shown that deviations from the norm in tooth morphology are not uncommon. Thus, a clear understanding of the root canal anatomy of the human dentition and its variations is a prerequisite for successful endodontic procedures.

The presence of two palatal roots in the maxillary molars, particularly in the second molars, is a rare phenomenon. Al Shalabi et al. [1], Green [2], and Vertucci [3] did not notice any maxillary second molars with two palatal root canals in their respective studies. Libfeld and Rotstein [4] reported a 0.4% incidence of four rooted maxillary second molars among 1200 teeth studied.

This paper describes two cases of unusual variation in root and canal morphology of four-rooted maxillary second molar with two buccal and two palatal canals and their endodontic management.

## 2. Case Report

### 2.1. Case 1

**Figure 1:**
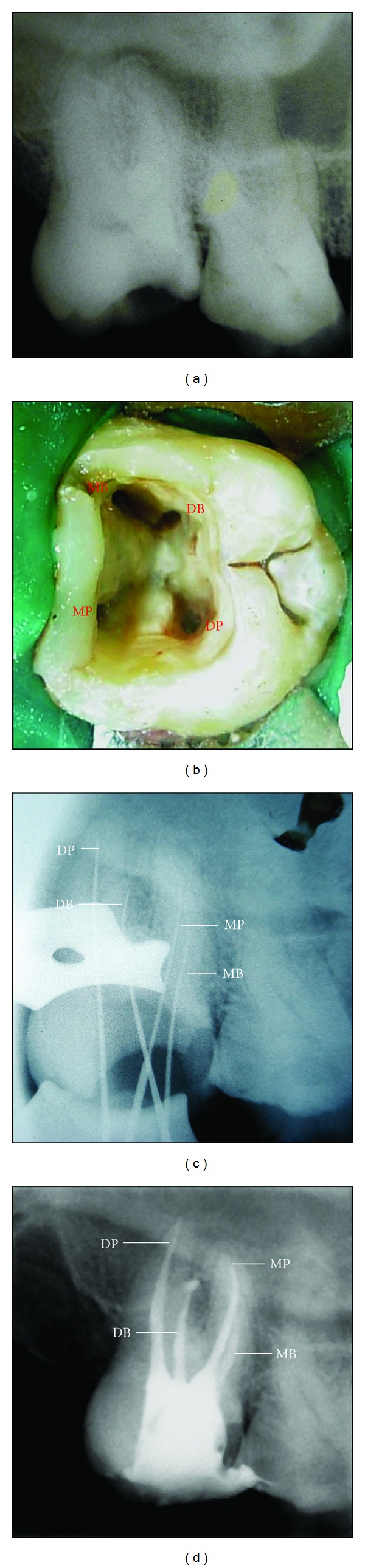
(a) Preoperative radiograph of the maxillary right second molar. (b) Access cavity revealing four distinct orifices. (c) Radiograph showing the working length of all four roots. (d) Postoperative radiograph of the obturated maxillary second molar.

A 37-year-old male with a noncontributory medical history was referred with a complaint of severe discomfort with his right maxillary teeth. The clinical and radiographic examinations revealed a maxillary right second molar with deep occlusal caries with tenderness on percussion. The clinical findings, radiographic findings, and pulp sensibility test led to a diagnosis of irreversible pulpitis with acute apical periodontitis with maxillary right second molar (Figure 1(a)), necessitating endodontic therapy. Radiographic evaluation of the involved tooth did not reveal any unusual anatomy.

The tooth was anesthetized and isolated with a rubber dam. The standard access opening was prepared with Cavity Access Set (Dentsply Maillefer, Ballaigues, Switzerland). Examination of the pulp chamber confirmed the presence of four orifices: two on the buccal aspect and two on the palatal aspect. Access cavity was modified from conventional triangular to square shape in order to achieve straight line access for all canals (Figure 1(b)).

The second palatal canal was explored with a DG-16 explorer and its presence was confirmed with an operating microscope. It was located mesial to the usual location of palatal canal and under the palatal aspect of the mesial marginal ridge. The mesiopalatal canal showed moderate apical curvature, while the disto-palatal canal was straight. The two palatal canal orifices were more widely placed as compared to the two buccal orifices. Thus the square formed by joining the imaginary lines connecting the four orifices was wider on palatal side. Radiographic examination revealed four separate roots with short height, positioned parallel to each other and blunt apices.

The pulp tissue was extirpated and working lengths were determined with an electronic apex locator (Root ZX, J. Morita Corp., Tokyo, Japan) and controlled with a periapical radiograph (Figure 1(c)). The four root canals were biomechanically prepared using crown-down technique with ProTaper NiTi rotary instruments (Dentsply Maillefer, Ballaigues, Switzerland). The canals were irrigated with 2.5% sodium hypochlorite and 17% EDTAC alternatively between each file during instrumentation.

At the second appointment (one week later), the root canals were obturated by the cold lateral condensation technique with gutta-percha cones and AH-Plus sealer (Dentsply De Trey GmbH, Konstanz, Germany) (Figure 1(d)). The tooth was restored with dual cure composite resin (LuxaCore Z, DMG, Germany).

### 2.2. Case 2

**Figure 2:**
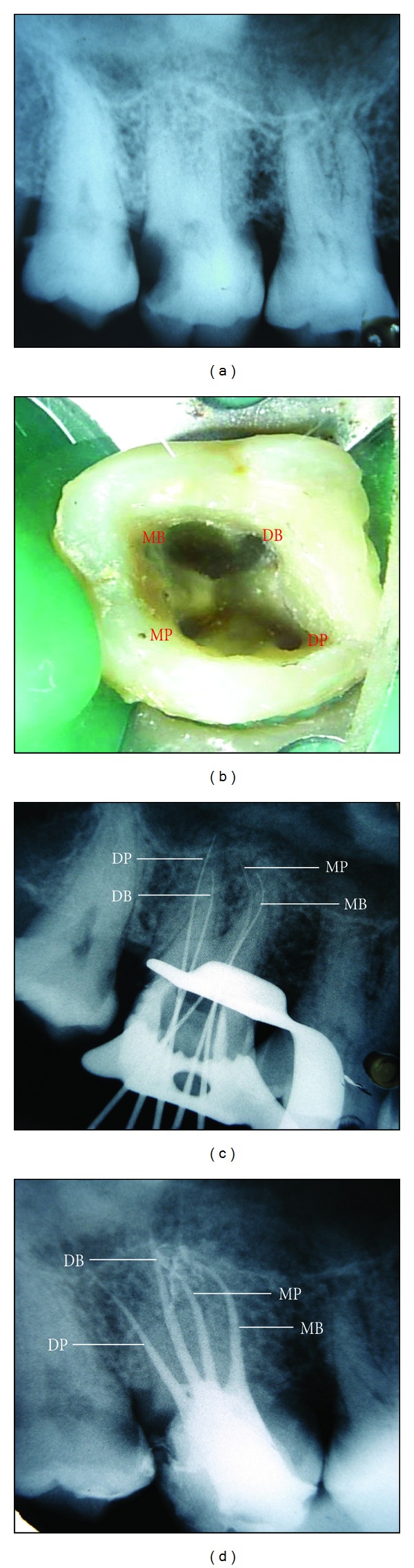
(a) Preoperative radiograph of the maxillary right second molar. (b) Access cavity revealing four distinct orifices. (c) Radiograph showing the working length of all four roots. (d) Postoperative radiograph of the obturated maxillary second molar.

A 48-year-old-male patient reported with a chief complaint of pain in the maxillary right posterior region. The clinical and radiographic examinations revealed a maxillary right second molar with deep disto-occlusal caries. The clinical and radiographic findings led to a diagnosis of chronic irreversible pulpitis with maxillary right second molar, necessitating endodontic therapy (Figure 2(a)).

The tooth was anesthetized and isolated with a rubber dam. The access opening was prepared which revealed four canal openings: two on the buccal and two on the palatal aspect (Figure 2(b)). The second palatal canal was explored with a DG-16 explorer and its presence was confirmed with an operating microscope. The access opening became square after preparation rather than conventional triangular shaped. Radiographic examination revealed four separate roots with short height, positioned parallel to each other and blunt apices.

Working length determination (Figure 2(c)), canal preparation, and obturation were done (Figure 2(d)) with the same materials and methods as described in the first case report and the tooth was restored with dual cure composite resin.

## 3. Discussion

The most common cause for the failure of root canal treatment is the incomplete removal of pulpal tissue apart from imperfect instrumentation and incomplete filling. This may occur due to the missing of anatomic aberration and/or extra canal during root canal procedure. Thus, thorough knowledge of the root canal system will help to reduce endodontic failures caused by incomplete debridement and obturation.

The incidence of four-rooted maxillary second molars is rare in the literature. Alavi et al. [6] investigated the root and canal morphologies of 268 maxillary molars in Thai population and failed to find any four-rooted maxillary molars. Peikoff et al. [7] observed that 1.4% of maxillary molars had second palatal root. In an *in vivo* study, Hartwell and Bellizzi [8] showed that 9.6% of the 176 maxillary second molars had four canals. However, the presence of two palatal roots has not been mentioned. Alani [9] described the endodontic treatment of bilateral maxillary second molars with 2 palatal roots.

Christie et al. [5] proposed a classification system for four-rooted maxillary second molar abnormalities depending on root separation level and divergence (Table 1). Sabala et al. [10] observed that the rarest aberrations (Type II palatal roots) are bilateral in 90% of cases. Baratto-Filho et al. [11] observed one palatal root with two distinct root canals, but it was fused with the mesiobuccal root up to the apical level. They suggested inclusion of this variety in the classification as Type IV.

Two cases reported in this paper revealed maxillary second molars with four separate roots of short height, positioned parallel to each other and blunt apices on radiographic examination, indicative of Type II maxillary second molar configuration.

The unusual anatomy of the maxillary second molar is difficult to diagnose because of its posterior location. Superimposition of the anatomical structures on the radiographs of this region may result in failure to diagnose a second palatal root canal. Exposing several radiographs from different angles may help to overcome the superimpositions and enable the clinician to identify this rare abnormality. Access to the root canal is the initial step in canal preparation. Properly designed and prepared access cavity will eliminate many potential problems during canal preparation and obturation. In the cases reported here, a large access was required to locate the two palatal canals. The access outline was square rather than conventional triangular. The two palatal canal orifices were more widely placed as compared to the two buccal orifices. Thus the square formed by joining the imaginary lines connecting the four orifices was wider on palatal side. Magnification aids like loupes and operating microscope should be used for a better visualization and location of anatomic aberrations.

When indistinct images of palatal roots are presented in preoperative X-ray images, the clinician must consider the possibility of two palatal roots. Dissociation of images must be performed and, if this anomaly is confirmed, a modified coronal access will allow the correct localization of root canals. Location and management of all anatomy is central to endodontic success.

## 4. Conclusion

Knowledge of possible variations in internal anatomy of human teeth is important for the successful outcome of endodontic treatment. A correct diagnosis and a careful clinical and radiographic inspection are required for endodontic success in teeth with a number of canals above that are normally found.

## Figures and Tables

**Table 1:** Classification of four-rooted maxillary second molar [5].

Type	Characteristics
I	Two widely divergent palatal roots that are often long and tortuous. Buccal roots of tooth are often cow horned and less divergent. Four separate root apices are seen on radiograph.
II	Four separate roots seen, but are often shorter, run parallel have buccal and lingual root morphology, and have blunt apices. Radiograph with buccolingual superimposition may make this appear as having only a mesial and distal root.
III	Constricted in root morphology with MB, MP, and DP canal encaged in a web of root dentin. The DB root in these cases appears to stand alone and may even diverge.
